# Cancer prevention with aspirin in hereditary colorectal cancer (Lynch syndrome), 10-year follow-up and registry-based 20-year data in the CAPP2 study: a double-blind, randomised, placebo-controlled trial

**DOI:** 10.1016/S0140-6736(20)30366-4

**Published:** 2020-06-13

**Authors:** John Burn, Harsh Sheth, Faye Elliott, Lynn Reed, Finlay Macrae, Jukka-Pekka Mecklin, Gabriela Möslein, Fiona E McRonald, Lucio Bertario, D Gareth Evans, Anne-Marie Gerdes, Judy W C Ho, Annika Lindblom, Patrick J Morrison, Jem Rashbass, Raj Ramesar, Toni Seppälä, Huw J W Thomas, Kirsi Pylvänäinen, Gillian M Borthwick, John C Mathers, D Timothy Bishop, CAPP2 Investigators, Alex Boussioutas, Carole Brewer, Jackie Cook, Diana Eccles, Anthony Ellis, Shirley V Hodgson, Jan Lubinski, Eamonn R Maher, Mary EM Porteous, Julian Sampson, Rodney J Scott, Lucy Side

**Affiliations:** a Translational and Clinical Research Institute, Newcastle University, Newcastle upon Tyne, UK; b Human Nutrition Research Centre, Population Health Sciences Institute, Newcastle University, Newcastle upon Tyne, UK; c Division of Haematology and Immunology, Leeds Institute of Medical Research at St James's, University of Leeds, Leeds, UK; d Colorectal Medicine and Genetics, Royal Melbourne Hospital, Melbourne, Australia; e Department of Education & Research, Jyväskylä Central Hospital, Jyväskylä, Finland; f Faculty of Sport and Health Sciences, University of Jyväskylä, Jyväskylä, Finland; g St Josefs-Hospital, Bochum-Linden, Germany; h National Cancer Registration and Analysis Service, Public Health England, London, UK; i Instituto Nazionale per lo Studio e, la Cura dei Tumori, Milan, Italy; j Division of Evolution and Genomic Medicine, University of Manchester, Manchester, UK; k St Mary's Hospital, Manchester Universities Foundation Trust, Manchester, UK; l Clinical Genetics, Rigshospital, Copenhagen, Denmark; m Hereditary GI Cancer Registry, Department of Surgery, Queen Mary Hospital, Hong Kong Special Administrative Region, China; n Department of Molecular Medicine & Surgery, Karolinska Institutet, Stockholm, Sweden; o Department of Medical Genetics, Queens University Belfast, Belfast City Hospital HSC Trust, Belfast, UK; p Genomic and Precision Medicine Research Unit, Division of Human Genetics, Institute of Infectious Diseases and Molecular Medicine, Faculty of Health Sciences, University of Cape Town, South Africa; q Department of Gastrointestinal Surgery, Helsinki University Hospital, Helsinki, Finland; r St Mark's Hospital, London, UK; s Faculty of Medicine, Imperial College London, London, UK

## Abstract

**Background:**

Lynch syndrome is associated with an increased risk of colorectal cancer and with a broader spectrum of cancers, especially endometrial cancer. In 2011, our group reported long-term cancer outcomes (mean follow-up 55·7 months [SD 31·4]) for participants with Lynch syndrome enrolled into a randomised trial of daily aspirin versus placebo. This report completes the planned 10-year follow-up to allow a longer-term assessment of the effect of taking regular aspirin in this high-risk population.

**Methods:**

In the double-blind, randomised CAPP2 trial, 861 patients from 43 international centres worldwide (707 [82%] from Europe, 112 [13%] from Australasia, 38 [4%] from Africa, and four [<1%] from The Americas) with Lynch syndrome were randomly assigned to receive 600 mg aspirin daily or placebo. Cancer outcomes were monitored for at least 10 years from recruitment with English, Finnish, and Welsh participants being monitored for up to 20 years. The primary endpoint was development of colorectal cancer. Analysis was by intention to treat and per protocol. The trial is registered with the ISRCTN registry, number ISRCTN59521990.

**Findings:**

Between January, 1999, and March, 2005, 937 eligible patients with Lynch syndrome, mean age 45 years, commenced treatment, of whom 861 agreed to be randomly assigned to the aspirin group or placebo; 427 (50%) participants received aspirin and 434 (50%) placebo. Participants were followed for a mean of 10 years approximating 8500 person-years. 40 (9%) of 427 participants who received aspirin developed colorectal cancer compared with 58 (13%) of 434 who received placebo. Intention-to-treat Cox proportional hazards analysis revealed a significantly reduced hazard ratio (HR) of 0·65 (95% CI 0·43–0·97; p=0·035) for aspirin versus placebo. Negative binomial regression to account for multiple primary events gave an incidence rate ratio of 0·58 (0·39–0·87; p=0·0085). Per-protocol analyses restricted to 509 who achieved 2 years' intervention gave an HR of 0·56 (0·34–0·91; p=0·019) and an incidence rate ratio of 0·50 (0·31–0·82; p=0·0057). Non-colorectal Lynch syndrome cancers were reported in 36 participants who received aspirin and 36 participants who received placebo. Intention-to-treat and per-protocol analyses showed no effect. For all Lynch syndrome cancers combined, the intention-to-treat analysis did not reach significance but per-protocol analysis showed significantly reduced overall risk for the aspirin group (HR=0·63, 0·43–0·92; p=0·018). Adverse events during the intervention phase between aspirin and placebo groups were similar, and no significant difference in compliance between intervention groups was observed for participants with complete intervention phase data; details reported previously.

**Interpretation:**

The case for prevention of colorectal cancer with aspirin in Lynch syndrome is supported by our results.

**Funding:**

Cancer Research UK, European Union, MRC, NIHR, Bayer Pharma AG, Barbour Foundation.

## Introduction

In 1988, Kune and colleagues reported an apparent protective effect of aspirin and other non-steroidal anti-inflammatory drugs in a colon cancer case-control study.^1^ In subsequent years, over 100 observational studies have confirmed the cancer preventive properties of aspirin.^2^, ^3^ A series of adenoma prevention trials reported a significant but modest effect of aspirin on new polyp formation.^4^ Reviews by Rothwell and colleagues of long-term cancer outcomes among participants in trials of the cardiovascular benefits of aspirin revealed a consistent pattern of significant reductions in cancer incidence and mortality among those randomly assigned to aspirin, following a delay of around 3–5 years.^5^, ^6^ The Women's Health Study^7^ randomly assigned over 18 000 women to alternate day 100 mg aspirin or vitamin E supplement. Intervention over 10 years showed no effect on cancer incidence. A subsequent post-trial review up to 18 years revealed a significantly lower incidence of colorectal cancer in those randomly assigned to aspirin.^8^

**Research in context**

**Evidence before this study**

There is extensive evidence from case-control studies, epidemiology, polyp prevention studies, and long-term review of historical aspirin cardiovascular prevention trials that regular intake of aspirin (acetyl salicylic acid) and other non-steroidal anti-inflammatory drugs over long periods is associated with reduced incidence of colorectal and other cancers. Two trials explored the protective effects of aspirin with cancer as a primary endpoint; the Women's Health Study showed no effect of alternate day 81 mg aspirin up to study end at 10 years, but a subsequent reduction in colorectal cancer in the second decade. The CAPP2 Study randomised 861 carriers of hereditary cancer, Lynch syndrome, average age 42 years, to 600 mg aspirin or 30 g resistant starch, or both in a factorial design. There was no effect at the end of intervention (average 2·5 years) but there was a significant per-protocol protective effect of the aspirin at an average of 4·6 years follow-up.

**Added value of this study**

The mechanisms of action of aspirin of relevance to colorectal cancer prevention, including for Lynch syndrome, are unknown, thus there is no insight into the time that the intervention takes to come into full effect or the period over which a time-limited intervention will allow protection. By monitoring people with known intervention and disease status, this study examines the magnitude of the effect and provides some indications as to the magnitude of the long-term effect.

The cancer histories of all participants in CAPP2 were reviewed where possible up to the planned 10-year follow-up and a subset in three countries was followed via national registries for up to 20 years. Aspirin prescription ended in 2007 at the latest. Intention-to-treat Cox proportional hazards analysis showed that aspirin protected against the primary endpoint of colorectal cancer (HR=0·65 [95% CI 0·43–0·97], p=0·035). Negative binomial regression, considering all primary cancer diagnoses in the cancer spectrum of Lynch syndrome found similar evidence of aspirin protection (incidence rate ratio 0·58, 95% CI 0·39–0·87, p=0·0085). For those who took aspirin for the planned minimum 2 years, the effect was similar; HR 0·56, CI 0·34–0·91, p=0·019.

There were half as many endometrial cancers in the aspirin group but, overall, non-colorectal cancer Lynch syndrome cancers were not significantly different in the longer follow-up. In the second decade, there was some evidence of a decline in non-Lynch syndrome cancers that did not reach significance.

**Implications of all the available evidence**

Two standard aspirins per day for 2 years results in a significant reduction in colorectal cancer incidence in Lynch syndrome carriers, which persists for over a decade but does not become apparent until about 5 years from commencement. During intervention, serious adverse events did not differ significantly from the placebo group in this relatively young group. The ongoing CaPP3 Study is a dose non-inferiority trial which will inform optimal doses for cancer prevention versus adverse events. There is now a strong case for prescribing aspirin to young adult carriers of a germline DNA mismatch repair gene defect.

The Cancer Prevention Programme (CaPP), originally called the Concerted Action Polyposis Prevention (CAPP) project, was launched in 1993 to investigate therapeutic prevention in those with a proven genetic predisposition to colorectal and other cancers. CAPP1^9^ focused on adolescents with familial adenomatous polyposis.^9^ CAPP2,^10^ which began in 1999, focused on people with Lynch syndrome, formerly known as hereditary non-polyposis colon cancer, which results from pathogenic variants in one of the DNA mismatch repair genes.^11^ In both trials, the potential cancer preventives were aspirin and resistant starch and a factorial design was used; in CAPP2, participants received 600 mg enteric-coated aspirin or placebo and 30 g of Novelose, a resistant starch, or placebo corn starch, daily. Recruits could opt to be randomly assigned for a single agent only. If a participant agreed to be randomly assigned for aspirin, recruits were treated for 2 years with aspirin or placebo; after a formal review, the design was adapted to allow recruits to consent to continue for a further 2-year period.

When participants had completed this intervention phase, data analysis revealed no significant impact on neoplasia which combined colorectal adenoma and carcinoma formation.^10^ At that stage, there were 20 diagnosed colorectal cancers.^10^ The protocol had anticipated a delayed effect on cancer incidence as is evident from epidemiological studies in the general population^12^ and provided for follow-up to 10 years. When the first recruits reached their 10-year follow-up (mean follow-up of 4 years 7 months), intention-to-treat analysis showed a non-significant protective effect on colorectal cancer in the aspirin limb compared with placebo^11^ (hazard ratio [HR]=0·63, 95% CI 0·35–1·13; p=0·12) with a similar result for all Lynch syndrome cancers. Per-protocol analysis was restricted to those who took aspirin or placebo for the defined minimum 2-year intervention period. This showed significant protection against colorectal cancer (HR=0·41, 95% CI 0·19–0·86; p=0·02) and a similar reduction for all Lynch syndrome cancers (HR=0·45, 95% CI 0·26–0·79; p=0·005).^11^ We now report the planned 10-year analysis, in which all participants were beyond the 10-year anniversary of their recruitment; this includes the period in which the participant was taking the agent. In addition, we complemented these data through access to the National Cancer Registration & Analysis Service via Public Health England, which provided robust cancer data for the English and Welsh recruits up to 20 years; equivalent data was obtained for the Finnish participants over the same time period.

## Methods

### Study design and participants

Between January, 1999, and March, 2005, 937 (87%) of 1071 assessed carriers of Lynch syndrome started intervention in the CAPP2 study. Eligible patients were older than 25 years of age and were proven carriers of a pathological mismatch-repair mutation (ie, genetic diagnosis) or were members of a family that met the Amsterdam diagnostic criteria and had a personal history of a cured Lynch syndrome neoplasm but a largely intact colon (ie, clinical diagnosis). Trial design and randomisation blocks have been described previously as has a detailed description of participant characteristics;^11^ a summary of some of the characteristics of the participants is shown in the appendix (p 8). All data relating to participants, their random assignment and all clinical outcomes in the trial were collected by the local clinical management centre for each participant and then sent to the CAPP2 Offices at Newcastle University, UK, where the dedicated CAPP2 study team maintained the information in a TrakGene database. These data were then linked to study number, anonymised, and sent to the University of Leeds (DTB and FE) for statistical analysis. Participants consented in writing to have their health records followed for 10 years. The study had a pre-planned design for 10-years' follow-up and at the time of this analysis, the last enrolled participant had reached the 10-year threshold. All participants consented to long-term follow-up at recruitment and consent was refreshed in the later stages of the study.

### Randomisation and masking

**Figure 1:**
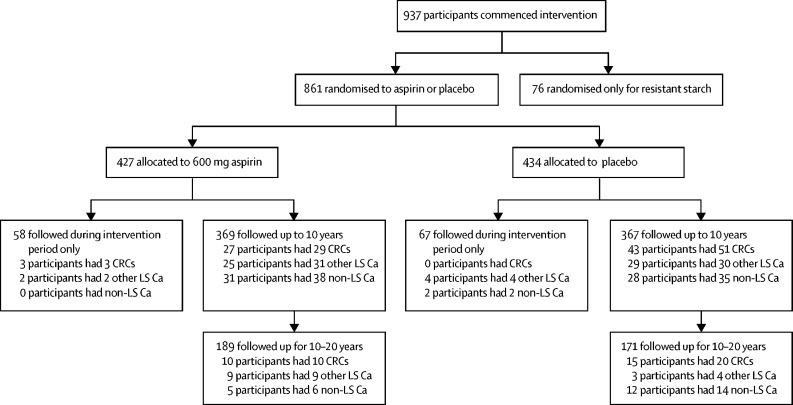
Trial profile CRC=colorectal cancer. LS Ca=Lynch syndrome associated cancers.

Briefly, of the 937 participants, 427 were randomly assigned to 600 mg aspirin and 434 to aspirin placebo (hereafter placebo; figure 1). The remaining recruits were not randomly assigned for the aspirin intervention, having opted not to participate in this part of the study (n=76, mostly owing to perceived aspirin sensitivity or history of peptic ulceration). All participants were also separately randomly assigned to resistant starch or resistant starch placebo intervention in a 2 × 2 factorial design. Details of the starch randomisation and outcomes were reported in 2012,^13^ and an update is in preparation (J Burn; personal communication). Participants and investigators were masked to intervention allocation. Participants and their clinicians were informed of their randomisation category at their 10-year review or later when contact was achieved.

### Procedures

Recruits received intervention over 2 years with an option to continue for a further 2 years. We also included data from analysis beyond the 10-year period and up to 20 years of follow-up for participants resident in England or Wales. For these participants, cancer history was assessed by means of national cancer registration information made available by Public Health England. Similarly, all participants in Finland are monitored centrally, and long-term cancer information was available for these participants from the Finnish Lynch syndrome registry. For the analysis between 10 and 20 years of follow-up, we combined information for 360 participants from England, Finland, and Wales.

Details of adenoma recurrence, adverse events during and post-intervention, and compliance (ie, proportion of scheduled tablets taken during the intervention phase) have been described previously.^11^

### Outcomes

The primary outcome of the CAPP2 study was the number, size, and histological stage of colorectal carcinomas found after a minimum of 2 years' intervention with aspirin. Secondary clinical outcomes were the size and number of adenomas and of other Lynch syndrome-related cancers.

This analysis focused on the 861 CAPP2 study participants randomly assigned to 600 mg aspirin (n=427) or placebo (n=434) from the date of entry until the latest date for which we had information about cancer status, often corresponding to the date of last surveillance attendance and, for recruits in England, Finland, and Wales, supplemented with passive recording of cancer registration. The analysis included Lynch syndrome cancer diagnoses; those that occurred during the intervention phase and subsequent to exit from the intervention phase. Designation of a reported diagnosis as being within the Lynch syndrome cancer spectrum was a clinical assessment, masked to intervention; endometrial, ovarian, pancreatic, small bowel, gall bladder, ureter, stomach, and kidney cancers and cancer of the brain were included.^14^ Adverse events during the intervention phase between aspirin and placebo groups were reported previously.^11^

### Statistical analysis

Power calculations suggested that to detect a 40% reduction in cancer risk among those taking aspirin with 90% power at a 0·05 significance level, about 100 cancer diagnoses post-recruitment were required, indicating approximately 1000 recruits and a 10-year follow-up. In total, 937 eligible patients with Lynch syndrome, mean age 45 years, commenced treatment, of which 861 agreed to be randomly assigned to the aspirin group or placebo and the mean intervention period was 2·5 years. When participants had completed this intervention phase, data analysis revealed no significant effect on neoplasia which combined colorectal adenoma and carcinoma formation.^10^ At that stage, there were 20 diagnosed colorectal cancers.^10^ The protocol had anticipated a delayed effect on cancer incidence as is evident from epidemiological studies in the general population^12^ and provided for follow-up to 10 years. When the first recruits reached their 10-year follow-up (mean follow-up of 4 years 7 months), intention-to-treat analysis showed a non-significant protective effect on colorectal cancer in the aspirin group compared with placebo with a similar result for all Lynch syndrome cancers.^11^ Per-protocol analysis was restricted to those who took aspirin or placebo for the defined minimum 2-year intervention period. This showed significant protection against colorectal cancer and a similar reduction for all Lynch syndrome cancers.^11^

Intention-to-treat analyses were done (ie, according to aspirin randomisation; aspirin or placebo). However, as the intention of this trial was to test the efficacy of 2-year intervention with aspirin, we also did a per-protocol analysis that was restricted to those participants who achieved the full 2-year intervention. Participants were provided with two 300 mg tablets daily over the first 2 years of the intervention phase and the numbers of aspirin (or placebo tablets) remaining were recorded. At clinic visits during the intervention period, each participant was asked to show the aspirin (or placebo) remaining, this number was recorded to allow estimation of the number of tablets taken. Participants whose study records indicated having taken at least 1400 tablets were considered as per protocol.

Two analytical approaches were used: time to first occurrence of colorectal cancer (our primary endpoint) was examined by means of life-table methods and Cox proportional hazards; and investigation of multiple primary cancers at the same anatomical site or multiple anatomical sites, a feature of Lynch syndrome, by means of negative binomial modelling which considers the complete Lynch syndrome cancer history since randomisation. Previously, we had applied Poisson regression but analysis of this extended dataset indicated over-dispersion, so we have applied negative binomial regression.^11^

For life-table analysis and Cox proportional hazards analysis, end of follow-up was determined as the time to first diagnosis of colorectal cancer (or other Lynch syndrome cancer, as appropriate), if the participant was diagnosed with cancer following random assignment, or the last recorded date at which clinical status was known. For the negative binomial regression analysis, exposure time was calculated from randomisation until the date of last known clinical status, or 10 years if this came earlier (20 years if resident in England, Finland, or Wales). Analyses were further done as intention to treat (ie, intervention assigned at randomisation) and per protocol. Beyond 10 years, cancer diagnosis follow-up data from England and Wales were obtained from Public Health England and their Welsh counterparts (see appendix p 3); all data released by Public Health England was with the approval of their Office for Data Release and within the policy and legal frameworks for the use of patient data collected by the National Cancer Registration and Analysis Service. Data from the Finnish national registry maintained by the Department of Surgery, Central Finland Healthcare District, Jyväskylä were accessed similarly (see appendix p 3).

Sex-adjusted and age-adjusted hazard ratio (HR) estimates and 95% CIs were calculated by means of Cox proportional hazard models, and Kaplan-Meier curves were used to assess non-parametrically the outcome differences between the aspirin and placebo groups; these adjustments were deemed desirable given the variation of cancer risk with age and the different spectrum of cancer in females and males. The assumption of proportional hazard was tested by means of Schoenfeld residuals. Sex-adjusted and age-adjusted incidence rate ratios were calculated by means of negative binomial regression to estimate the effect of aspirin from log-linear models for the number of primary cancers diagnosed after randomisation and until the last follow-up.

A secondary analysis examined the incidence of non-colorectal Lynch syndrome cancers. A final analysis examined the total burden of Lynch syndrome-related cancers. In keeping with the original sample size calculations, all p values were two-sided. All analyses were carried out in Stata (version 14).

The trial is registered with the ISRCTN registry, number ISRCTN59521990.

### Role of the funding source

Neither the funders nor the sponsors of the study had any role in study design, data collection, data analysis, data interpretation, or writing of the report. The corresponding authors had full access to all the data in the study and had final responsibility for the decision to submit for publication.

## Results

Between January, 1999, and March, 2005, 937 carriers of Lynch syndrome in England, Finland, and Wales of 1071 assessed started intervention in the CAPP2 study. Table 1 documents the characteristics of the 427 CAPP2 participants randomly assigned to aspirin and the 434 participants randomly assigned to placebo. Each group had approximately 25 months of intervention and a mean of more than 7 years' follow-up post-intervention. The distribution of time since recruitment was also similar between the groups (table 1); 58 (14%) of the aspirin group and 67 (15%) of the placebo group had no follow-up after the intervention phase, predominantly because of refusal to consent to long-term follow-up (61%) or loss to follow-up (34%; figure 1). Since randomisation, 40 (9%) of 427 participants randomly assigned to aspirin developed colorectal cancer compared with 58 (13%) of 434 allocated to placebo (table 1). When all Lynch syndrome cancers are included, 74 (17%) of 427 participants from the aspirin group had developed cancer compared with 89 (21%) of 434 in the placebo group (table 1).

**Table 1:** The whole CAPP2 cohort at 10 years plus England, Finland, and Wales registry data to 20 years

		**Aspirin (n=427)**	**Placebo (n=434)**	**Total (n=861)**
Time in CAPP2 intervention study (months)*		25·0 (12·5, 0·8–60·6)	25·4 (14·2, 1·1–74·4)	25·2 (13·4, 0·8–74.4)
Months between study entry and last known follow-up date*		120·4 (63·3, 1·6–238·7)	116·3 (63·7, 1·1–238·9)	118·4 (63·5, 1·1–238·9)
Years between study entry and last known follow-up date				
	≤2	36 (8%)	42 (10%)	78 (9%)
	>2 and ≤6	80 (19%)	87 (20%)	167 (19%)
	>6 and ≤10	133 (31%)	144 (33%)	277 (32%)
	>10 and ≤14	54 (13%)	43 (10%)	97 (11%)
	>14 and ≤18	104 (24%)	101 (23%)	205 (24%)
	>18 and ≤20	20 (5%)	17 (4%)	37 (4%)
Participants with first colorectal cancer				
	Since randomisation	40 (9%)	58 (13%)	98 (11%)
	Within 2 years of randomisation	10 (2%)	10 (2%)	20 (2%)
	More than 2 years from randomisation	30 (7%)	48 (11%)	78 (9%)
Participants with other Lynch syndrome cancers (excluding colorectal)				
	Since randomisation	36 (8%)	36 (8%)	72 (8%)
	Within 2 years of randomisation	7 (2%)	9 (2%)	16 (2%)
	More than 2 years from randomisation	29 (7%)	27 (6%)	56 (7%)
Participants with one or more Lynch syndrome cancers (including colorectal)				
	Since randomisation	74 (17%)	89 (21%)	163 (19%)
	Within 2 years of randomisation	17 (4%)	19 (4%)	36 (4%)
	More than 2 years from randomisation	57 (13%)	70 (16%)	127 (15%)
Types of extracolonic Lynch syndrome cancers				
	Brain	2 (<1%)	0	2 (<1%)
	Stomach, duodenum	5 (1%)	6 (1%)	11 (1%)
	Bile duct, pancreas	8 (2%)	3 (1%)	11 (1%)
	Urinary†	7 (2%)	6 (1%)	13 (2%)
	Ovarian	2 (<1%)	2 (<1%)	4 (<1%)
	Endometrium–uterine	8 (2%)	17 (4%)	25 (3%)
	Multiple sites	4 (1%)	2 (<1%)	6 (1%)
Participants with non-Lynch syndrome cancers				
	Since randomisation	36 (8%)	42 (10%)	78 (9%)
	Within 2 years of randomisation	2 (<1%)	7 (2%)	9 (1%)
	More than 2 years from randomisation	34 (8%)	35 (8%)	69 (8%)

* Data are mean (SD, range) or n (%).

† Urinary cancers include ureter and kidney cancers.

We present analyses of the combined information from the post-trial surveillance of participants for the first 10 years across all centres with that collected for up to 20 years from national cancer data registry data sources for England (58%), Finland (41%), and Wales (1%). Information on the separate analyses of these datasets can be found in the appendix. For completeness, the numbers are reported for the overall 10-year follow-up of all participants in the appendix (p 9) and for the 20-year follow-up involving participants in England, Finland, and Wales only in the appendix (p 12). The close alignment between data collected by the CAPP Centres in the first 10 years of follow-up for England, Finland, and Wales and the information collected directly from national health data sources for the same time period are shown in the appendix (p 11). There was almost complete concordance in reporting of colorectal cancer diagnoses between the CAPP centre and the national sources; however, concordance for both non-colorectal cancer Lynch syndrome diagnoses and non-Lynch syndrome diagnoses was lower, most notably for Finland. These observations reflect the fact that most recruiting centres took responsibility for organising and reviewing bowel surveillance while the spectrum of non-colorectal cancers challenged the capabilities of some centres to obtain complete information.

**Figure 2:**
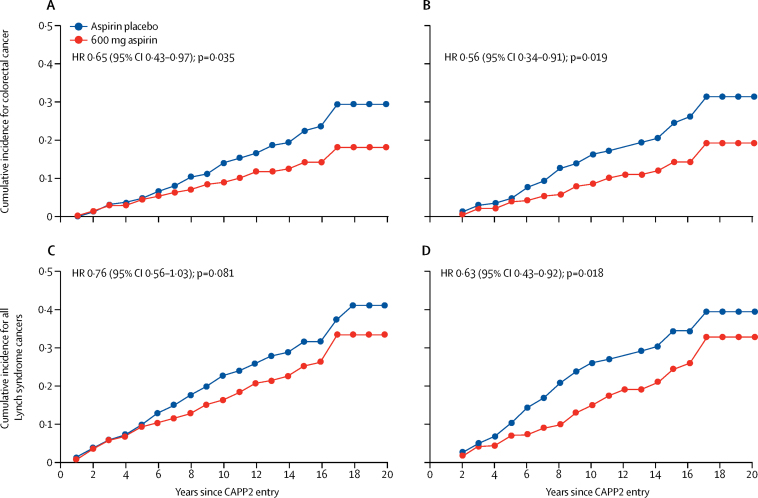
Time to first colorectal cancer and time to any Lynch syndrome cancer in all CAPP2 study participants followed up for 10 years and for 20 years in England, Finland, and Wales Cox proportional hazards (HRs and 95% CIs) comparing those on aspirin *vs* those on placebo and depicted by Kaplan-Meier analysis (n=861). (A) Intention-to-treat analysis (n=427 aspirin, 434 placebo) by randomisation group. (B) Per-protocol analysis of all those achieving 2 years aspirin or placebo (n=259 aspirin; n=250 placebo). (C) Intention-to-treat analysis for any Lynch syndrome cancer. (D) Per-protocol analysis for any Lynch syndrome cancer. See appendix (p 16) for more details.

Intention-to treat analysis showed a reduced HR of 0·65 (95% CI 0·43–0·97; p=0·035) for aspirin versus placebo (table 2; figure 2A; appendix p 16). Per-protocol analysis restricted to 509 participants who had achieved a minimum of 2 years on treatment showed a significantly reduced HR of 0·56 (0·34–0·91; p=0·019; table 2; figure 2B; appendix p 16). Since some participants were diagnosed with multiple primary cancers, we did an incidence rate ratio analysis by means of negative binomial regression, which showed similar estimates for effect sizes and significance levels to the time to first colorectal cancer analysis (table 1).

**Table 2:** Cox proportional hazards and negative binomial regression analyses of cancer incidence*

		**Hazard ratio**†**(95% CI)**	**p value**	**Incidence rate ratio**‡**(95% CI)**	**p value**
**Colorectal cancer**					
Intention-to-treat analysis (n=861, 98 events for hazard ratio analysis)					
	Aspirin *vs* placebo	0·65 (0·43–0·97)	0·035	0·58 (0·39–0·87)	0·0085
Per-protocol analysis§ (n=509, 67 events)					
	≥2 years' placebo	1·0	..	1·0	..
	≥2 years' aspirin	0·56 (0·34–0·91)	0·019	0·50 (0·31–0·82)	0·0057
Cumulative aspirin dose¶ (n=861, 98 events)					
	Units of 100 aspirin	0·98 (0·96–1·00)	0·079	0·98 (0·96–1·00)	0·032
**Non-colorectal Lynch syndrome cancers**					
Intention-to-treat analysis (n=861, 72 events)					
	Aspirin *vs* placebo	0·94 (0·59–1·50)	0·81	1·05 (0·65–1·69)	0·84
Per-protocol analysis§ (n=509, 46 events)					
	≥2 years' placebo	1·0	..	1·0	..
	≥2 years' aspirin	0·75 (0·42–1·34)	0·33	0·87 (0·48–1·61)	0·67
Cumulative aspirin dose¶ (n=861, 72 events)					
	Units of 100 aspirin	0·98 (0·96–1·01)	0·20	0·99 (0·97-1·02)	0·50
**All Lynch syndrome cancers**					
Intention-to-treat analysis (n=861, 163 events)					
	Aspirin *vs* placebo	0·76 (0·56–1·03)	0·081	0·75 (0·56–1·02)	0·065
Per-protocol analysis§ (n=509, 107 events)					
	≥2 years' placebo	1·0	..	1·0	..
	≥2 years' aspirin	0·63 (0·43–0·92)	0·018	0·65 (0·44–0·94)	0·022
Cumulative aspirin dose¶ (n=861, 163 events)					
	Units of 100 aspirin	0·98 (0·97–1·00)	0·033	0·98 (0·97–1·00)	0·040
**All non-Lynch syndrome cancers**					
Intention-to-treat analysis (n=861, 78 events)					
	Aspirin *vs* placebo	0·81 (0·52–1·26)	0·34	0·79 (0·49–1·28)	0·34
Per-protocol analysis§ (n=509, 56 events)					
	≥2 years' placebo	1·0	..	1·0	..
	≥2 years' aspirin	0·81 (0·48–1·37)	0·43	0·71 (0·41–1·22)	0·21
Cumulative aspirin dose¶ (n=861, 78 events)					
	Units of 100 aspirin	0·99 (0·97–1·01)	0·43	0·99 (0·96–1·01)	0·32

* Adjusted for age and gender in all participants up to 10 years and up to 20 years in England, Finland, and Wales, randomly assigned to aspirin or placebo.

† Adjusted for age at consent and gender.

‡ Incidence rate ratio from negative binomial regression adjusted for age at consent and gender.

§ The threshold for 2 years' intervention was consumption of more than 1400 aspirin tablets; rounded from a 2-year total of 1461 to allow for early scheduling of the exit colonoscopy or occasional missed dosage.

¶ Units of 100 aspirin=total number of aspirin taken divided by 100.

Separate 10-year and 20-year analyses are reported in the appendix (pp 10, 13, 17–18) with similar findings with respect to effect sizes.

For non-colorectal Lynch syndrome cancers, neither intention-to-treat analysis (HR=0·94, 95% CI 0·59–1·50); p=0·81) nor per-protocol analysis (HR=0·75, 0·42–1·34; p=0·33; table 2) showed any significant effect of aspirin chemoprevention.

Separate 10-year and 20-year analyses are also reported in the appendix (pp 10, 13, 17–18); for this analysis, there is less consistency in the estimates between the analyses but there is no evidence of a cancer-prevention effect overall. Examination of individual anatomical sites is not feasible given the numbers of cases. However, for endometrial cancer, the most common Lynch syndrome cancer after the colorectum, the observation of only seven cases among women on aspirin compared with 17 on placebo is suggestive but not conclusive of a protective effect of aspirin (HR=0·50, 95% CI 0·22–1·11; p=0·09; appendix p 19).

For all Lynch syndrome cancers, intention-to-treat analysis showed no significant effect (HR=0·76, 95% CI 0·56–1·03; p=0·081) with similar results for the incidence rate ratio analysis. However, per-protocol analysis showed reduced risk among aspirin takers (HR=0·63, 0·43–0·92; p=0·018) with similar estimates and significance for the incidence rate ratio analysis (table 2; appendix p 16). Intention-to-treat and per-protocol analysis for non-colorectal Lynch syndrome cancers are shown in the appendix (p 14) and the results for the separate 10-year and 20-year analyses are shown in the appendix (pp 17–18).

Although evidence of the beneficial effect of aspirin on cancer risk seems strongest for colorectal cancer, there is some evidence of a broader spectrum of activity^2^ so we examined the effect of aspirin on risk of all non-Lynch syndrome cancers (details in appendix p 14). There was no significant evidence of any effect of aspirin on non-Lynch syndrome cancer risk (appendix p 10). In the data available for the second decade, there were five cancers in the aspirin group and 12 in the placebo group (HR=0·56, 95% CI 0·27–1·19; p=0·13; appendix p 14).

We investigated whether aspirin treatment affected the stage at which cancers were diagnosed. For the 54 tumours for which Dukes' stage information was available, there was no evidence of any stage differences between those randomly assigned to aspirin and to placebo (p=0·37; appendix p 15).

## Discussion

Evidence accumulated over 30 years shows that frequent aspirin ingestion reduces cancer risk in the general population;^15^, ^16^ analysis of data from observational studies, from trials with cardiovascular disease as the primary endpoint and from one trial with cancer as an endpoint, shows that the protective effect of aspirin takes 3–10 years to become apparent in the general population. Follow-up for 25 years and more in these studies showed no evidence that the protective effect of aspirin had disappeared although there was evidence of a reduced effect of low doses in people of greater bodyweight.^17^

This analysis of data from people with Lynch syndrome recruited to the CAPP2 study shows that the protective effects of aspirin against colorectal cancer has persisted in this long-term analysis of up to 20 years' follow-up. The CAPP2 study data were analysed at the end of the intervention, again when the first recruits reached their 10-year follow-up and now when all participants reached 10-years' follow-up and some approached 20 years since recruitment. Colorectal cancer incidence began to diverge about 5 years after initiating aspirin treatment and this lower cancer incidence was maintained throughout the period of observation. This pattern is similar to that observed in the long-term follow-up of participants randomly assigned to aspirin in cardiovascular disease prevention trials.^12^, ^18^

This long-term follow-up of cancer among patients confirms the view that 600 mg aspirin per day reduces the risk of colorectal cancer in patients with Lynch syndrome. While we had shown the protective effect in per protocol analysis (ie, restricting the focus to those who had regularly taken the aspirin during the intervention phase),^11^ this analysis confirms the extended effect both in the per-protocol group but, now, also in the intention-to-treat analysis of all participants. As would be expected, the effect sizes in the intention-to-treat analysis are less extreme than the per-protocol analysis. The support from this intention-to-treat analysis reduces any concerns that the previous per-protocol finding was attributable, not to aspirin, but to an unidentified confounding factor which associated with non-adherence.

Whereas the evidence for CRC chemoprevention with aspirin for the genetically predisposed has increased, the evidence for prevention at the other anatomical sites relevant to Lynch syndrome has weakened.

The mechanism of action of aspirin in cancer prevention remains to be established; the beneficial effect of a range of NSAIDs in cancer prevention and the association between PIK3CA mutation and response of colorectal cancers to aspirin use in the Nurses' Health Study^19^ point to a long-term influence of the suppression of inflammation. The benefits seen in the Women's Health Study^8^ followed use of alternate day low-dose aspirin, however, which is too low to suppress inflammation directly.

The response to aspirin might reflect an effect on the viability of cells with malignant potential as suggested by studies in cell lines and mouse models.^20^, ^21^ Salicylates in plants modulate the apoptotic response to infection^22^ and might have a similar pro-apoptotic influence in the gut.^23^, ^24^ Enhanced destruction of premalignant abnormal cells would offer an explanation for the long time-lag in response to aspirin intervention. The progressive decline in mitochondrial function, on which apoptosis is reliant, in the gut in old age might then contribute to the lesser response to aspirin chemoprevention in old age.^25^, ^26^ The widespread occurrence of aberrant colonic crypts, defective in mismatch repair, in Lynch syndrome^27^ suggests that these might be the premalignant lesions that are suppressed or destroyed by long-term aspirin exposure, but this hypothesis remains to be tested. In addition, whether this occurs via enhanced apoptosis^22^ or improved immune surveillance,^28^ or a combination of these and other mechanisms are important areas for future investigation.

It has been suggested that the results of the first follow-up analysis might have reflected a temporary delay in progression of premalignant lesions rather than a true prevention of cancer. This is not supported by the continued separation of the cumulative incidence of colorectal cancer in the two treatment groups.

It is noteworthy that the effect on non-colorectal Lynch syndrome cancers was of similar magnitude to the lower intestinal effects in the 10-year data. The benefit in prevention of non-colorectal cancer tumours was not apparent in the data available for the second decade, suggesting that any preventive effect is not sustained. This is in keeping with the long-term follow up of the Women's Health Study^8^ in which the effect of low dose aspirin, which appeared at 10 years, was evident for colorectal cancer only.

Weaknesses of this trial are that the geographically dispersed recruitment over a long period made collection of details of endoscopy difficult, so data on adenomas are incomplete and the current analysis is restricted to cancer as an endpoint. We were unable to comment on effect on mortality as the death rate in patients with Lynch syndrome undergoing surveillance is low; that would require a much larger study. Our study attempted to recruit patients with a confirmed, significant mutation in a mismatch repair gene. On that basis, we suggest that the finding is generalisable across all patients with Lynch syndrome. However, we do not have the power to indicate if particular subgroups (eg specific genes or mutations) have effect sizes different from the population overall. Side-effects of treatment were minimal in this relatively young population but are likely to be much higher if aspirin continues to be used into old age as illustrated by the ASPREE trial.^29^ Conversely, the report of an overall anticancer benefit in people over 65 years of age^30^ is a reminder of the potential long-term benefits of aspirin as a means of therapeutic prevention of cancer in the wider population. It is noteworthy that 427 participants had 18 fewer colorectal cancers as a result of taking two aspirins a day for an average of 2·5 years, a number needed to treat of 24.

The CAPP2 study tested the effect of a single dose of aspirin (600 mg per day) and this study provides evidence that this dose was effective in long-term prevention of colorectal cancer. However, whether this is the optimum aspirin dose in respect of the balance between benefit (cancer risk reduction) and risk (gastrointestinal bleeding) remains to be determined. To address the issue of dosage, CaPP3 is a randomised dose non-inferiority trial of the dose of 600 mg daily, compared with 300 mg and 100 mg, delivered in a blinded fashion for 2 years followed by open-label treatment for a further 3 years and assessment of Lynch syndrome cancers annually thereafter.

With a minimum target of 1500 mismatch repair gene defect carriers, the study closed to recruitment on March 31, 2019, having randomly assigned a total of 1882 Lynch syndrome gene carriers in the UK and four other countries. The first analysis of data from the randomised trial will be done in 2024.

In conclusion, the data reported here support the recommendation that adult carriers of a pathogenic mismatch repair gene defect (Lynch syndrome) should be advised that taking 600 mg aspirin daily for at least 2 years significantly reduces the risk of a future cancer, bearing in mind that this effect does not become apparent for at least 4 years.

## Data sharing

For further access to data, please contact the corresponding author (JB).
