# Supplementary material for: Cancer prevention with aspirin in hereditary colorectal cancer (Lynch syndrome), 10-year follow-up and registry-based 20-year data in the CAPP2 study: a double-blind, randomised, placebo-controlled trial
Source: Lancet. 2020 Jun 13;395(10240):1855–63. doi: 10.1016/S0140-6736(20)30366-4 (PMC7294238; doi:10.1016/S0140-6736(20)30366-4)

THE LANCET

Supplementary appendix

This appendix formed part of the original submission and has been peer reviewed. We post it as supplied by the authors.

Supplement to: Burn J, Sheth H, Elliott F, et al. Cancer prevention with aspirin in hereditary colorectal cancer (Lynch syndrome), 10-year follow-up and registry-based 20-year data in the CAPP2 study: a double-blind, randomised, placebo-controlled trial. *Lancet* 2020; **395:** 1855–63.

Double Blind Randomised Placebo Controlled Trial Of Cancer Prevention With Aspirin In Hereditary Colorectal Cancer (Lynch Syndrome): Planned 10 Year Follow-Up And Registry Based 20 Year Data In The CAPP2 Study

John Burn MD, Harsh Sheth PhD, Faye Elliott MSc, Lynn Reed, Finlay Macrae MD, Jukka-Pekka Mecklin MD, Gabriela Möslein MD, Fiona E. McRonald PhD, Lucio Bertario MD, D. Gareth Evans MD, Anne-Marie Gerdes MD, Judy W.C. Ho MD, Annika Lindblom MD, Patrick J. Morrison MD, Jem Rashbass MD, Raj Ramesar PhD, Toni Seppälä MD, Huw J. W. Thomas MD, Kirsi Pylvänäinen, Gillian M. Borthwick PhD, John C. Mathers PhD, D Timothy Bishop PhD, on behalf of the CAPP2 Investigators

Contents

1. Supplementary Note
    a. Statistical Methods: p 3
    b. Principal Investigators: p 7
2. Supplementary Table S1, Baseline Characteristics: p 8
3. Supplementary Table S2, Characteristics of total CAPP2 Cohort at 10 year follow-up: p 9
4. Supplementary Table S3, Analysis of total CAPP2 Cohort at 10 year follow-up: p 10
5. Supplementary Table S4, Comparison of CAPP2 records and national cancer registration: p 11
6. Supplementary Table S5, Characteristics of English, Finnish and Welsh participants at 20 years: p 12
7. Supplementary Table S6, Analysis of English, Finnish and Welsh participants to 20 years: p 13
8. Supplementary Table S7, Non-LS cancers occurring during study: p 14
9. Supplementary Table S8, Dukes stage by randomisation group: p 15
10. Supplementary Figure S1, HR & IRR analysis for cancer incidence for total Cohort over total study: p 16
11. Supplementary Figure S2, HR & IRR analysis for cancer incidence for total Cohort over first 10 years: p 17
12. Supplementary Figure S3, HR & IRR analysis for English, Finnish and Welsh participants to 20 years: p 18
13. Supplementary Figure S4, Kaplan-Meier graph of time to first endometrial cancer: p 19

Supplementary Note

Statistical Methods

Long-term data from England and Wales

NCRAS provided information on all diagnoses of cancer among the CAPP2 cohort in England and Wales through to 2017; these data were obtained from Public Health England (PHE). This provides ICD-coded information on specific diagnoses and provides dates of registration etc. For inclusion in the life-table analyses, we needed to confirm that participants had remained resident in England or Wales throughout this time period (whether or not they had a cancer diagnosis) so we supplemented this information with identification of current General Practitioner for each participant to confirm UK residence. On that basis, we assumed complete cancer information for these participants (i.e. that absence of a diagnosis meant that participant was cancer-free for that time period) through to the last date at which the Registry was considered complete. Long term follow-up was considered known up to this time point except for those participants who were known to have died or emigrated in which case their follow-up was censored at death or at estimated date of emigration. Supplementary Table 3 compares the information from PHE with that from the CAPP2 Trial database.

Long-term data from Finland

Similar data (to that provided for England and Wales) were available from the Lynch Syndrome Registry of Finland (LSRFi) maintained by the Department of Surgery, Jyväskylä, Finland. The registry works as a national research database of MMR pathogenic variant carriers and their high-risk family members. Historically, the first families have been included in the registry based on clustered LS-associated cancers within the families, and later on as the family variants were identified. After genetic testing became available, only pathogenic variants are recorded in the registry. At present, families are identified either by universal MMR testing of LS-related cancers or by clinical phenotype.

Within Finland, most centres report their clinical findings to the LSRFi office routinely. The LSRFi office keeps track of scheduled surveillance for each carrier and requests the clinical reports from the treating institutions regularly based on the national study permission of the LS research group. The third line of confirmation for full cancer information is the nationally operated Finnish Cancer Registry that is queried for the LSRFi PV carriers regularly/when needed. Supplementary Table 4 compares the information from Finnish LS Registry with that from the CAPP2 Trial database.

The administration and the ethical board of the Central Finland health care district approved the study.

Supplementary Table 1 shows descriptive statistics of baseline characteristics in all 861 participants, then in three subgroups; 125 participants followed to the end of intervention, 376 participants followed from start of intervention to 10th anniversary and 360 participants followed from start of intervention to 20th anniversary. There were no differences in age, sex, eligibility status and MMR mutation status between the three groups of participants.

Ten Year Analysis of All CAPP2 Participants

Supplementary Table 2 documents the characteristics of the 427 CAPP2 participants randomised to aspirin and the 434 participants to placebo. Characteristics of the two groups are very similar with approximately 25 months of intervention in each group and mean >7 years follow-up post-intervention. The distribution of time since recruitment was also similar between the groups (Supplementary Table 2). Since randomisation, 30 participants randomised to aspirin developed CRC compared with 43 allocated to placebo. Supplementary Table 2 documents the number of CRCs and LS cancers. When all LS cancers were included, 55 participants from the aspirin group had developed cancer compared with 73 in the placebo group (Supplementary Table 2).

Intention to treat (ITT) analysis showed a non-significantly reduced hazard ratio (HR) of 0.68 (95% CI 0.42-1.08), p=0.10 for aspirin versus placebo (Supplementary Table 3; Supplementary Figure 2A). Per protocol (PP) analysis restricted to participants who had achieved a minimum of two years on treatment (n=509) showed a significantly reduced hazard ratio of 0.52 (95% CI 0.29-0.94), p=0.029 (Supplementary Table 3; Supplementary Figure 2B). Since some

participants were diagnosed with multiple primary cancers, we conducted an IRR analysis using a negative binomial analysis which showed similar estimates for effect sizes and significance levels to the time to first CRC analysis (Supplementary Table 3). For non-CRC LS cancers, neither ITT analysis (HR = 0.78 (95% CI 0.47-1.30) p=0.34) nor per protocol analysis (HR = 0.56 (95% CI 0.29-1.07), p = 0.080) (Supplementary Table 3) showed any significant effect of aspirin.

For all LS cancers, ITT analysis also showed no significant effect (HR = 0.72; 95% CI 0.50 – 1.02), p = 0.061 with similar results for the IRR analysis. However, PP analysis showed reduced risk among aspirin takers (HR = 0.53 (95% CI 0.34-0.83), p = 0.0050 with similar estimates and significance for the IRR analysis (Supplementary Table 3; Supplementary Figures 2E, 2F). Supplementary Figures 2C and 2D show ITT and PP analysis for non-CRC LS cancers. While evidence for the beneficial effect of aspirin on cancer risk is strongest for CRC, there is some evidence of a broader spectrum of activity [2] so we examined the effect of aspirin on risk by of all non-LS cancers (Supplementary Table 3). There was no evidence of any effect of aspirin on non-LS cancer risk (Supplementary Table 3, Supplementary Figures 2G, 2H)).

Twenty Year Analysis of data from participants in England, Finland and Wales

Supplementary Table 4 documents the diagnoses recorded in the CAPP2 Study TrakGene database as well as those obtained through access from PHE (England and Wales) plus from Finland. Separately, for England and Wales, and then for Finland, we compared diagnoses recorded in the corresponding datasets. There were three discrepancies for CRC between the CAPP2 database across all 3 countries but there were more differences for non-CRC cancers and non-LS cancers, likely reflecting the screening priorities among the participating centres for LS patients and the long term nature of this study. In each instance, the diagnostic records were accurate so the dataset utilized in the analysis consists of the combination of all records of diagnoses.

Supplementary Table 5 documents the characteristics of the 211 English and Welsh and 149 Finnish participants for whom complete cancer information was available (n= 360 total, comprising 42% of CAPP2 Study participants included in the 10 year analysis described above) and involving 189 participants randomised to aspirin and 171 to placebo. Mean follow-up for this analysis was 14 years 9 months. In this time period, 27 and 41 participants randomised to aspirin and placebo, respectively, developed CRC and there were 24 and 17 non-CRC LS cancers, respectively.

In the ITT analysis (Supplementary Table 6, Supplementary Figure 3A), CRC incidence was lower in those randomised to aspirin (HR = 0.58 (95% CI 0.36-0.94) p = 0.029) and the IRR analysis (which considers the number of cancers beyond the first one) confirmed this effect (HR = 0.50 (95% CI 0.31-0.80) p = 0.0039). PP analysis showed stronger evidence of a protective effect (Cox proportional hazard: HR = 0.43 (95% CI 0.23-0.79) p = 0.0063; IRR = 0.40 (95% CI 0.22-0.72) p = 0.0021) (Supplementary Table 6, Supplementary Figure 3B).

As expected, analysis of all LS cancers showed effect sizes and significance intermediate between the results for CRC alone and for non-CRC LS cancers (Supplementary Table 6, Supplementary Figure 3E). PP analysis showed fewer LS cancers in those randomised to aspirin (HR=0.57 (95%CI 0.36-0.92) p=0.021) (Supplementary Figure 3F). Supplementary Figures 3C and 3D show the ITT and PP analyses for non-CRC LS cancers with no discernible effect on cancer incidence following aspirin. However, for non-LS cancer there was a suggestion of a protective effect of aspirin (ITT analysis HR = 0.54 (95% CI 0.29-1.00) p = 0.051) in the 20 year follow-up of participants resident in England, Finland and Wales. The effect was similar in the PP analysis (HR=0.56 (95%CI 0.27-1.19) p=0.13) (Supplementary Figures 3G and 3H).

Principal Investigators:

Other CAPP2 Investigators (in addition to those named as co-authors of this manuscript):

Prof Alex Boussioutas MD, University of Melbourne, Peter MacCallum Cancer Centre, Royal Melbourne Hospitals, Melbourne, Australia

Dr Carole Brewer MD, Royal Devon and Exeter NHS Foundation Trust, Exeter, UK

Dr Jackie Cook, MD Sheffield Children's Hospital, Sheffield Children's NHS Foundation Trust, Sheffield, UK

Prof Diana Eccles MD, Faculty of Medicine, University of Southampton, Southampton, UK

Dr Anthony Ellis MD, Department of Medicine, University of Liverpool & Department of Gastroenterology, Royal Liverpool University Hospital, Liverpool, UK

Prof Shirley V Hodgson MD, Emeritus, St Georges, London, UK

Prof Jan Lubinski MD, Department of Genetics and Pathology, Pomeranian Medical University, Szczecin, PL

Prof Eamonn R Maher MD, University of Cambridge, NIHR Cambridge Biomedical Research Centre, Cancer Research UK Cambridge Biomedical Campus, Cambridge, UK

Dr Mary EM Porteous MD, University of Edinburgh, SE Scotland Genetics Service, Edinburgh, UK

Prof Julian Sampson MD, Division of Cancer and Genetics, School of Medicine, Cardiff University, Cardiff, UK

Prof Rodney J Scott PhD, Discipline of Medical Genetics, University of Newcastle, NSW, Australia & Hunter Medical Research Institute & Division of Molecular Medicine, NSW Health Pathology North, John Hunter Hospital, NSW, Australia

Dr Lucy Side MD, Wessex Clinical Genetics Service, University Hospitals Southampton, UK

Supplementary Table S1: Table of baseline characteristics

Variable	Study population (N=861)	Followed to the end of intervention (n=125)	Followed from start of intervention to 10th anniversary (n=376)	Followed from start of intervention to 20th anniversary (n=360)
**Age at study entry – yr**				
Mean (s.d)	44.3 (10.6)	43.5 (10.9)	44.0 (10.4)	44.9 (10.7)
Range	21.5-77.9	21.5-77.9	24.7-75.5	24.6-75.5
**Sex no. (%)**				
Female	476 (55.3)	67 (53.6)	210 (55.9)	199 (55.3)
Male	385 (44.7)	58 (46.4)	166 (44.1)	161 (44.7)
**Geographic region no. (%)**				
England/Wales	212 (24.6)	1 (0.8)	0 (0)	211 (58.6)
Finland	149 (17.3)	0 (0)	0 (0)	149 (41.4)
Other Northern Europe	250 (29.0)	66 (52.8)	184 (48.9)	0 (0)
Scotland/Ireland	43 (5.0)	13 (10.4)	30 (8.0)	0 (0)
Australia and Hong Kong	112 (13.0)	19 (15.2)	93 (24.7)	0 (0)
Southern Europe	53 (6.2)	23 (18.4)	30 (8.0)	0 (0)
South Africa	38 (4.4)	2 (1.6)	36 (9.6)	0 (0)
Americas	4 (0.5)	1 (0.8)	3 (0.8)	0 (0)
**Eligibility status at recruitment no. (%)**				
Clinical diagnosis	139 (16.1)	22 (17.6)	60 (16.0)	57 (15.8)
Genetic diagnosis	722 (83.9)	103 (82.4)	316 (84.0)	303 (84.2)
**Mutation no./total no. (%)**				
MLH1	433/722 (60.0)	61/103 (59.2)	163/316 (51.6)	209/303 (69.0)
MSH2	264/722 (36.5)	40/103 (38.8)	136/316 (43.0)	88/303 (29.0)
MSH6	25/722 (3.5)	2/103 (1.9)	17/316 (5.4)	6/303 (2.0)

Supplementary Table S2: Characteristics of CAPP2 Study participants at 10 years follow-up

	**Aspirin (n=427)**	**Placebo (n=434)**	**Total (n=861)**
**Time in CAPP2 intervention study (months)***	25.0 (12.5, 0.8-60.6)	25.4 (14.2, 1.1-74.4)	25.2 (13.4, 0.8-74.4)
**Months between study entry and last known follow-up date ***	94.5 (38.0, 1.6-120)	92.3 (38.8, 1.1-120)	93.4 (38.4, 1.1-120)
**Years between study entry and last known follow-up date (n)**			
⩽2	36	42	78
>2 and ⩽4	33	29	62
>4 and ⩽6	47	58	105
>6 and ⩽8	25	29	54
>8 and ⩽10	286	276	562
**Participants with first colorectal cancer (n)**			
Since randomisation	30	43	73
Within 2 years of randomisation	10	10	20
More than 2 years from randomisation	20	33	53
**Participants with other LS cancers (excluding colorectal) (n)**			
Since randomisation	27	33	60
Within 2 years of randomisation	7	9	16
More than 2 years from randomisation	20	24	44
**Participants with one or more LS cancer (including colorectal) (n)**			
Since randomisation	55	73	128
Within 2 years of randomisation	17	19	36
More than 2 years from randomisation	38	54	92
**Types of extra-colonic LS cancers** (n)**			
Brain	3	0	3
Upper GI			
Stomach	2	3	5
Duodenum	2	3	5
Bile duct	2	0	2
Pancreas	6	3	9
Urinary[+]	6	5	11
Ovarian	4	3	7
Endometrium	8	17	25
**Participants with non-LS cancers (n)**			
Since randomisation	31	30	61
Within 2 years of randomisation	2	7	9
More than 2 years from randomisation	29	23	52

* Data are mean (SD; range)

** 4 participants in aspirin and 1 participant in placebo had more than one extra-colonic LS cancer

[+] Urinary cancers include ureter and kidney cancer

Supplementary Table S3: Cox proportional hazards and negative binomial regression analysis (adjusted for age and gender) for colorectal cancer, non-colorectal LS cancers, all LS cancers and non-LS cancers in participants randomly assigned to aspirin or placebo at 10 years follow-up

	**Hazard ratio (HR) (95% CI) ***	**p value**	**Incidence rate ratio (95% CI) †**	**p value**
**Colorectal Cancer**				
Intention-to-treat analysis (N=861, 73 events in HR analysis)				
Aspirin versus placebo	0.68 (0.42-1.08)	0.10	0.64 (0.40-1.02)	0.061
Per-Protocol Analysis‡ (N=509, 49 events)				
⩾2 years' placebo	1.0	-	1.0	-
⩾2 years' aspirin	0.52 (0.29-0.94)	0.029	0.49 (0.27-0.88)	0.018
Cumulative aspirin dose§ (N=861, 73 events)				
Units of 100 aspirin	0.98 (0.95-1.01)	0.12	0.98 (0.95-1.00)	0.087
**Non-colorectal LS cancers**				
Intention-to-treat analysis (N=861, 60 events)				
Aspirin versus placebo	0.78 (0.47-1.30)	0.34	0.89 (0.53-1.50)	0.66
Per-Protocol Analysis‡ (N=509, 39 events)				
⩾2 years' placebo	1.0	-	1.0	-
⩾2 years' aspirin	0.56 (0.29-1.07)	0.080	0.69 (0.36-1.33)	0.27
Cumulative aspirin dose§ (N=861, 60 events)				
Units of 100 aspirin	0.98 (0.95-1.01)	0.14	0.99 (0.96-1.01)	0.30
**All LS cancers**				
Intention-to-treat analysis (N=861, 128 events)				
Aspirin versus placebo	0.72 (0.50-1.02)	0.061	0.75 (0.53-1.06)	0.099
Per-Protocol Analysis‡ (N=509, 84 events)				
⩾2 years' placebo	1.0	-	1.0	-
⩾2 years' aspirin	0.53 (0.34-0.83)	0.0050	0.58 (0.38-0.90)	0.015
Cumulative aspirin dose§ (N=861, 128 events)				
Units of 100 aspirin	0.98 (0.96-1.00)	0.034	0.98 (0.96-1.00)	0.055
**All non-LS cancers**				
Intention-to-treat analysis (N=861, 61 events)				
Aspirin versus placebo	0.96 (0.58-1.59)	0.89	0.99 (0.58-1.70)	0.98
Per-Protocol Analysis‡ (N=509, 45 events)				
⩾2 years' placebo	1.0	-	1.0	-
⩾2 years' aspirin	0.96 (0.54-1.73)	0.90	0.87 (0.48-1.57)	0.65
Cumulative aspirin dose§ (N=861, 61 events)				
Units of 100 aspirin	1.00 (0.97-1.03)	0.98	1.00 (0.97-1.03)	0.82

*adjusted for age at consent and gender

† Incidence rate ratio from negative binomial regression adjusted for age at consent and gender

‡ The threshold for 2 years' intervention was consumption of more than 1400 aspirin tablets; rounded from a 2-year total of 1461 to allow for early scheduling of the exit colonoscopy or occasional missed dosage.

§ Units of 100 aspirin= total number of aspirin taken divided by 100.

Supplementary Table S4: Follow-up information CAPP2 Study participants resident in England and Wales was collected by the CAPP2 Study TrakGene database as well as obtained from the National Cancer Registry & Analysis Service for England plus its Welsh counterpart and made available for analysis by Public Health England (PHE). The concordance between the CAPP2 database and PHE for England/Wales is shown in the top Table with corresponding information for Finland in the lower Table. The top table shows the diagnoses which are consistent between the CAPP2 database and the PHE records (except for modest differences in dates of diagnosis), those which were known to the CAPP2 database but were not found in NCRAS system and then those which were found in the NCRAS system but were not known to the CAPP2 database with the diagnosis being in a time period that the CAPP2 database covered. The final row ("Identified by PHE after last follow-up..") reflect diagnoses made after the last active follow-up for the CAPP2 database. Similar information is available for Finland.

**English and Welsh Records in participants randomised to aspirin or placebo**	**First CRC**	**First LS cancer (excluding CRC)**	**First Non-LS cancer**
Consistent between CAPP2 Study database and PHE	24	8	10
Reported by CAPP2 Study database; record not found by PHE in NCRAS system	1	0	1
Identified by PHE only between study entry and last follow-up date known to CAPP2 study	0	3	2
Identified by PHE after last follow-up date known to CAPP2 study	15	13	19
**Total**	**40**	**24**	**32**
**% concordance**	**24/25=96%**	**8/11=73%**	**10/13=77%**

**Finnish Records in participants randomised to aspirin or placebo**	**First CRC**	**First LS cancer (excluding CRC)**	**First Non-LS cancer**
Consistent between CAPP2 Study database and Finnish records	15	5	5
Reported by CAPP2 Study database; record not found by in Finnish clinical records	0	0	0
Identified by Finnish records only between study entry and last follow-up date known to CAPP2 study	2	6	5
Identified by Finnish records after last follow-up date known to CAPP2 study	11	6	1
**Total**	**28**	**17**	**11**
**% concordance**	**15/17=88%**	**5/11=45%**	**5/10=50%**

Supplementary Table S5: Characteristics of the English, Welsh and Finnish participants in the CAPP2 Study with up to 20 years follow-up

	Aspirin (n=189)	Placebo (n=171)	Total (n=360)
**Time in CAPP2 intervention study (months)***	26.3 (13.5, 0.8-54.7)	26.8 (14.3, 2.1-74.4)	26.6 (13.9, 0.8-74.4)
**Months between study entry and last known follow-up date***	175.5 (38.2, 24.1-238.7)	178.4 (36.2, 42.6-238.9)	176.9 (37.2, 24.1-238.9)
**Years between study entry and last known follow-up date (n)**			
≤2	0	0	0
>2 and ≤6	6	4	10
>6 and ≤10	5	6	11
>10 and ≤14	54	43	97
>14 and ≤18	104	101	205
>18 and ≤20	20	17	37
**Participants with first colorectal cancer (n)**			
Since randomisation	27	41	68
Within 2 years of randomisation	6	4	10
More than 2 years from randomisation	21	37	58
**Participants with other LS cancers (excluding colorectal) (n)**			
Since randomisation	24	17	41
Within 2 years of randomisation	3	3	6
More than 2 years from randomisation	21	14	35
**Participants with one or more LS cancer (including colorectal) (n)**			
Since randomisation	49	55	104
Within 2 years of randomisation	9	7	16
More than 2 years from randomisation	40	48	88
**Types of extra-colonic LS cancers** (n)**			
Brain	1	0	1
Upper GI			
Stomach	2	2	4
Duodenum	3	2	5
Bile duct	2	0	2
Pancreas	5	2	7
Urinary+	6	4	10
Ovarian	2	1	3
Endometrium	5	7	12
**Participants with non-LS cancers (n)**			
Since randomisation	16	27	43
Within 2 years of randomisation	0	1	1
More than 2 years from randomisation	16	26	42

* Data are mean (SD; range). ** 2 participants in aspirin and 1 participant in placebo had more than one extra-colonic LS cancer

+ Urinary cancers include ureter and kidney cancer

Supplementary Table S6: Cox proportional hazards and negative binomial regression analysis (adjusted for age and gender) for colorectal cancer, non-colorectal LS cancers, all LS cancers and non-LS cancers in English, Welsh and Finnish population with up to 20 years follow-up randomly assigned to aspirin or placebo.

	**Hazard ratio (HR)* (95% CI)**	**p value**	**Incidence rate ratio† (95% CI)**	**p value**
**Colorectal Cancer**				
Intention-to-treat analysis (N=360, 68 events in HR analysis)				
Aspirin versus placebo	0.58 (0.36-0.94)	0.029	0.50 (0.31-0.80)	0.0039
Per-Protocol Analysis‡ (N=231, 47 events)				
⩾2 years' placebo	1.0	-	1.0	-
⩾2 years' aspirin	0.43 (0.23-0.79)	0.0063	0.40 (0.22-0.72)	0.0021
Cumulative aspirin dose§ (N=360, 68 events)				
Units of 100 aspirin	0.97 (0.95-0.99)	0.038	0.97 (0.94-0.99)	0.011
**Non-colorectal LS cancers**				
Intention-to-treat analysis (N=360, 41 events)				
Aspirin versus placebo	1.23 (0.66-2.29)	0.51	1.28 (0.70-2.35)	0.43
Per-Protocol Analysis‡ (N=231, 28 events)				
⩾2 years' placebo	1.0	-	1.0	-
⩾2 years' aspirin	0.99 (0.47-2.10)	0.98	1.00 (0.48-2.06)	1.00
Cumulative aspirin dose§ (N=360, 41 events)				
Units of 100 aspirin	0.99 (0.96-1.02)	0.49	0.99 (0.96-1.02)	0.51
**All LS cancers**				
Intention-to-treat analysis (N=360, 104 events)				
Aspirin versus placebo	0.75 (0.51-1.11)	0.15	0.70 (0.49-1.01)	0.056
Per-Protocol Analysis‡ (N=231, 71 events)				
⩾2 years' placebo	1.0	-	1.0	-
⩾2 years' aspirin	0.57 (0.36-0.92)	0.021	0.56 (0.36-0.88)	0.010
Cumulative aspirin dose§ (N=360, 104 events)				
Units of 100 aspirin	0.98 (0.96-1.00)	0.038	0.98 (0.96-0.99)	0.014
**All non-LS cancers**				
Intention to treat analysis (N=360, 43 events)				
Aspirin versus placebo	0.54 (0.29-1.00)	0.051	0.54 (0.30-0.98)	0.045
Per-Protocol Analysis‡ (N=231, 30 events)				
⩾2 years' placebo	1.0	-	1.0	-
⩾2 years' aspirin	0.56 (0.27-1.19)	0.13	0.56 (0.27-1.15)	0.11
Cumulative aspirin dose§ (N=360, 43 events)				
Units of 100 aspirin	0.97 (0.94-1.00)	0.071	0.97 (0.94-1.00)	0.091

* adjusted for age at consent and gender

† Incidence rate ratio from negative binomial regression adjusted for age at consent and gender

‡ The threshold for 2 years' intervention was consumption of more than 1400 aspirin tablets; rounded from a 2-year total of 1461

to allow for early scheduling of the exit colonoscopy or occasional missed dosage.
§ Units of 100 aspirin= total number of aspirin taken divided by 100.

Supplementary Table S7: Table of non-LS cancers occurring in CAPP2 10 year follow-up within England, Finland and Wales in the first decade since randomisation and also the second decade as well as all other Centres in the first decade. Data obtained from CAPP2 Database as well as PHE for England and Wales and Finnish clinical records.

Type of non-LS cancer[1]	England, Finland and Wales 1-10 years		Other Centres[2] 1-10 years		Total - All Centres 1-10 years			England, Finland and Wales 10-20 years	
	Aspirin	Placebo	Aspirin	Placebo	Aspirin	Placebo		Aspirin	Placebo
**Bladder**	1	2	1	1	2	3		1	0
**Bone**	0	0	1	0	1	0		0	0
**Breast**	2	3	4	2	6	5		0	2
**Cervix**	1	0	0	0	1	0		0	0
**Chondrosarcoma**	0	0	1	0	1	0		0	0
**Connective tissue**	0	0	0	1	0	1		0	0
**Leukaemia**	0	0	1	0	1	0		0	0
**Lung**	1	0	0	0	1	0		0	3
**Lymphoma**	0	0	1	1	1	1		0	0
**Meningioma**	0	0	0	1	0	1		0	0
**Neurofibrosarcoma**	0	0	0	1	0	1		0	0
**Oesophagus**	0	1	0	1	0	2		0	1
**Prostate**	2	3	5	2	7	5		0	3
**Skin**	4	5	4	5	8	10		4	3
**Thyroid**	0	1	0	0	0	1		0	0
**Tongue**	0	0	1	0	1	0		0	0
**Urethra**	0	0	1	0	1	0		0	0
**Total**	**11**	**15**	**20**	**15**	**31**	**30**		**5**	**12**

[1]Type of first non-LS cancer post study entry. [2] Centres excluding England, Finland and Wales

Supplementary Table S8: Dukes stage by randomisation group. Data were available on 62 colorectal tumours at 10 years with no evidence of any difference between the two groups (p = 0.37 Fisher exact after combining C and D into a single stage).

Dukes Stage	Placebo	Aspirin 600 mg	Total
**A**	17 (45%)	14 (58%)	**31**
**B**	16 (42%)	6 (25%)	**22**
**C**	4 (10%)	4 (17%)	**8**
**D**	1 (3%)	0 (0%)	**1**
**TOTAL**	**38 (100%)**	**24 (100%)**	**62**

Supplementary Figure S1: Time to first colorectal cancer and time to any LS cancer in CAPP2 study participants from England, Finland and Wales with up to 20 years follow-up and from the remaining centres with up to 10 years follow-up analysed using Cox proportional hazards (HR and 95% confidence intervals) comparing those on aspirin versus those on placebo and depicted by Kaplan-Meier analysis. The figures also show the number of participants at risk by time on study as well as 95% confidence intervals for the cumulative incidence of cancer. Figure 1A shows the intention to treat analysis (n=427 aspirin, 434 placebo) by randomisation group. Figure 1B shows the per protocol analysis of all those achieving 2 years aspirin or placebo (n=259 aspirin; n=250 placebo). Figure 1C shows the intention to treat analysis for LS cancer other than CRC and Figure 1D shows the per protocol analysis for this same comparison. Figures 1E and 1F show the results for ITT and per protocol analysis of all LS cancers combined. Finally, Figures 1G and 1H show the results for all non-LS cancers.

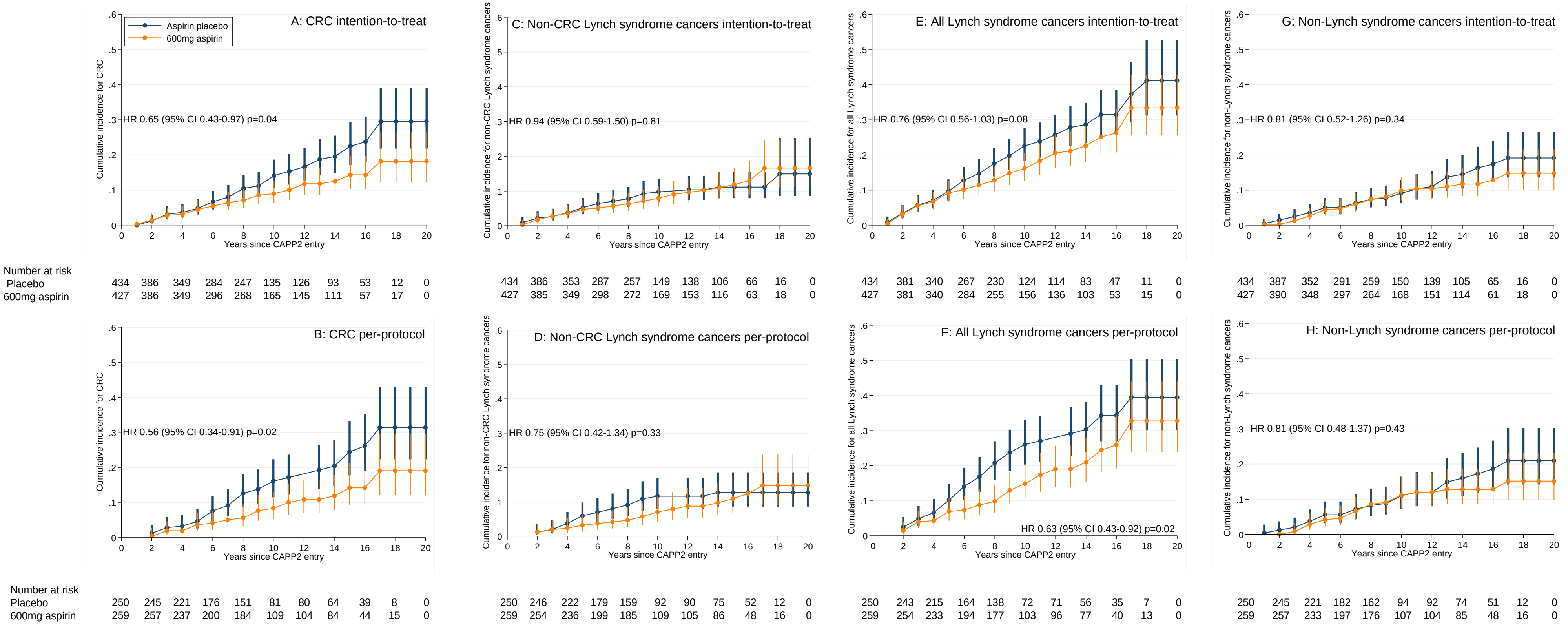

Supplementary Figure S2: Time to first colorectal cancer and time to any LS cancer in CAPP2 Study participants (n=861) for the first ten years after randomisation analysed using Cox proportional hazards (HR and 95% confidence intervals) comparing those on aspirin versus those on placebo and depicted by Kaplan-Meier analysis. The figures also show the number of participants at risk by time on study as well as 95% confidence intervals for the cumulative incidence of cancer. Figure 2A shows the intention to treat analysis (n=427 aspirin, 434 placebo) by randomisation group. Figure 2B shows the per protocol analysis of all those achieving 2 years aspirin or placebo (n=259 aspirin; n=250 placebo). Figure 2C shows the intention to treat analysis for LS cancer other than CRC and Figure 2D shows the per protocol analysis for this same comparison. Figures 2E and 2F show the results for ITT and per protocol analysis of all LS cancers combined. Finally, Figures 2G and 2H show the results for all non-LS cancers.

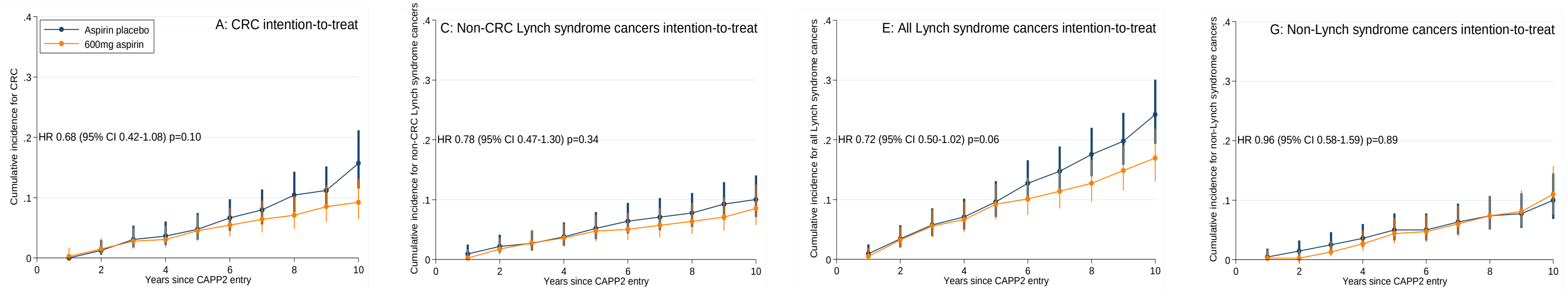

Number at risk	A						C						E						G					
Placebo	434	386	349	284	247	0	434	386	353	287	257	0	434	381	340	267	230	0	434	387	352	291	259	0
600mg aspirin	427	386	349	296	268	0	427	385	349	298	272	0	427	381	340	284	255	0	427	390	348	297	264	0

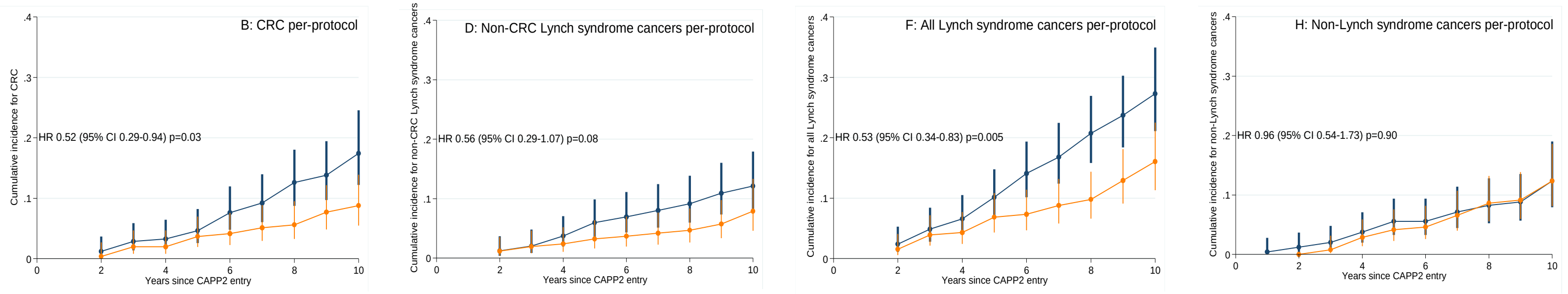

Number at risk	B						D						F						H					
Placebo	250	245	221	176	151	0	250	246	222	179	159	0	250	243	215	164	138	0	250	245	221	182	162	0
600mg aspirin	259	257	237	200	184	0	259	254	236	199	185	0	259	254	233	194	177	0	259	257	233	197	176	0

Supplementary Figure S3: Time to first colorectal cancer and time to any LS cancer in CAPP2 Study participants from England, Wales and Finland (n=360; 211 England and Wales, 149 Finland) for the first twenty years after randomisation analysed using Cox proportional hazards (HR and 95% confidence intervals) comparing those on aspirin versus those on placebo and depicted by Kaplan-Meier analysis. The lifetables show the number of persons at risk by time and study and 95% confidence intervals for cumulative incidence. Figure 3A shows the intention to treat analysis (n=189 aspirin; n=171 placebo) by randomisation group. Figure 3B shows the per protocol analysis of all those achieving 2 years aspirin or placebo (n=121 aspirin; n=110 placebo). Figure 3C shows the intention to treat analysis for LS cancer other than CRC and Figure 3D shows the per protocol analysis for this same comparison. Figures 3E and 3F show the results for ITT and per protocol analysis of all LS cancers combined. Finally, Figures 3G and 3H show the results for all non-LS cancers.

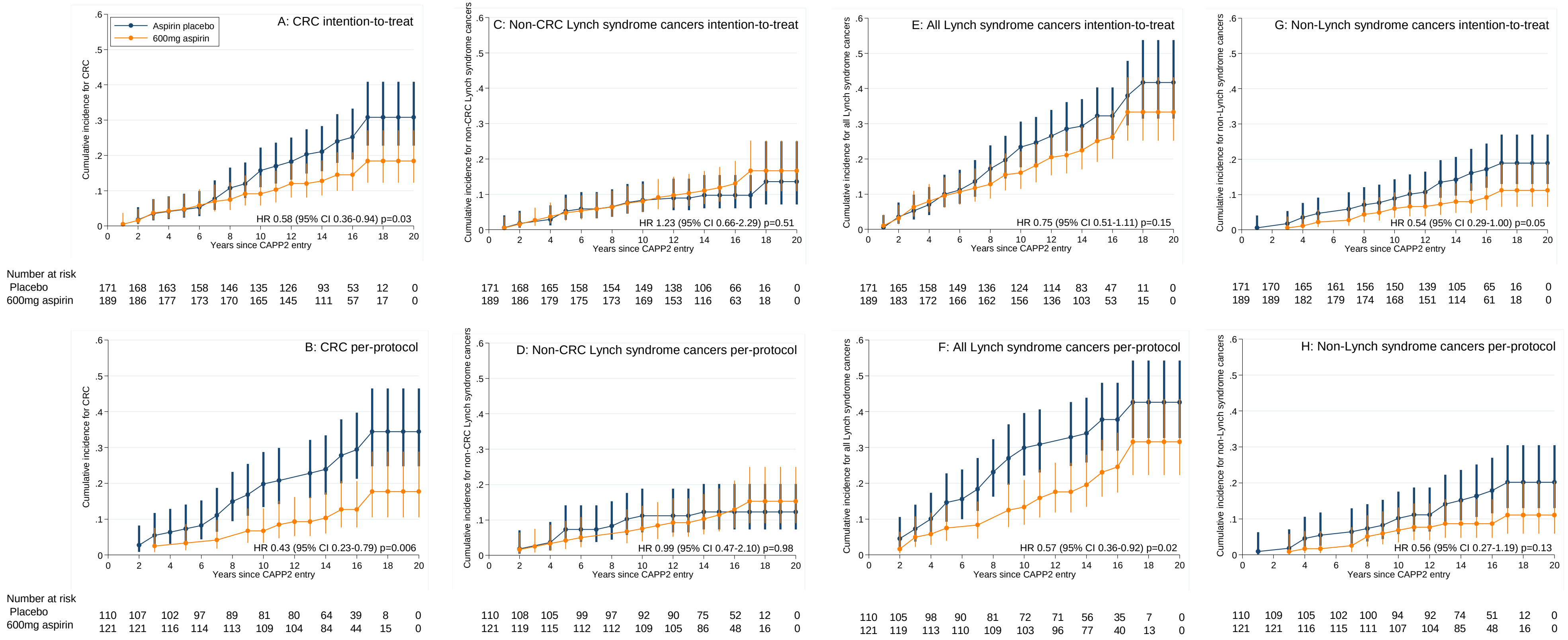

Supplementary Figure S4: Lifetable analysis of time until first endometrial cancer after recruitment among female CAPP2 participants. The format is as for previous figures.

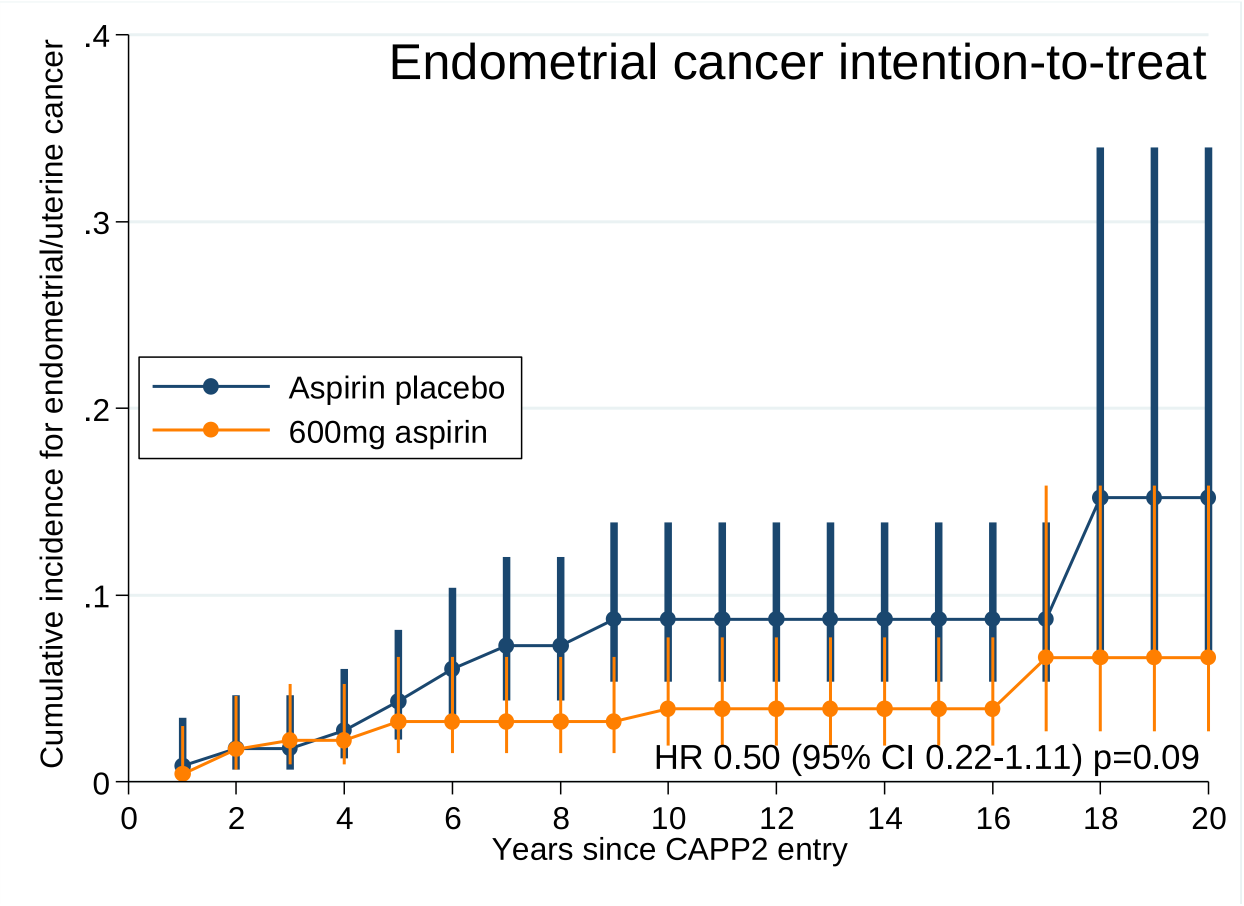

Supplement: Supplementary appendix [file mmc1.pdf]
